# Towards Better Understanding of the Harmful Impact of Hindrance and Challenge Stressors on Job Burnout of Nurses. A One-Year Cross-Lagged Study on Mediation Role of Work-Family Conflict

**DOI:** 10.3389/fpsyg.2021.696891

**Published:** 2021-09-17

**Authors:** Łukasz Baka, Monika Prusik

**Affiliations:** ^1^The Maria Grzegorzewska University, Warsaw, Poland; ^2^University of Warsaw, Warsaw, Poland

**Keywords:** hindrance and challenge stressors, job burnout, work-family conflict, nurses, mediation effect

## Abstract

The mediation role of work–family conflict (WFC) in job demands – job burnout link is well documented, also in group of nurses. It is still unclear, however, which job demands are particularly conducive to WFC and job burnout. Moreover the mediational effect of WFC was tested mainly in cross-sectional studies that were conducted in countries of North America and Western Europe. Drawing on the Job Demands-Resources and the Effort-Recovery models, this one-year cross-lagged study investigates the effects of five types of job demands related to challenge and hindrance stressors on job burnout (measured with exhaustion and disengagement from work) as well as the mediational role of WFC in Polish nurses. Job demands included emotional, cognitive demands, and demands for hiding emotions (as challenge stressors) as well as quantitative demands and work pace (as hindrance stressors). Data were collected among 516 nurses. Structural equation modelling (SEM) showed that hindrance stressors (T1) are predictor of higher job burnout (T2). The positive role of challenge stressors (T1) were not supported. Only emotional demands were associated with exhaustion but the direction of the relation was opposite than expected. WFC (T1) mediated the harmful effect of the two hindrance stressors and emotional demands on disengagement from work (but not on exhaustion). Cognitive demands and demands for hiding emotions were not related to negative outcomes. The obtained results shed light on the role of the challenge-hindrance stressors and WFI in development of job burnout. The implications for theory and research on the mental health of nurses are discussed.

## Introduction

As representatives of social service, nurses are especially vulnerable to job burnout (Maslach et al., 2001). According to the Job Demands-Resources model (Bakker and Demerouti, 2017), job burnout is a response to a prolonged state of imbalance between job demands and job resources. The direct relation between job demands and job burnout is very well documented (e.g., Schaufeli and Bakker, 2004; Alarcon, 2011), also in groups of nurses (Hansen et al., 2009; Jourdain and Chênevert, 2010; Garrosa et al., 2011; Adriaenssens et al., 2015; Dall’Ora et al., 2020); therefore, researchers are looking for potential mediational variables that would deepen the understanding of mechanisms regulating this relationship. Considering the irregular work hours of nurses, the regular night shifts and the fact that nursing is an occupation dominated by women, who tend to have greater family responsibilities than men (Mayrhofer et al., 2008; Maher, 2013), the work-family conflict (WFC) is pointed out as a key mediator of the job demands –burnout link in this profession (Unruh et al., 2016; Gonnelli and Raffagnino, 2017). The mediation effect of WFC is confirmed in groups of nurses but mainly in cross-sectional (not cross-lagged) studies that were conducted in countries of North America and Western Europe (Janssen et al., 2004; Leineweber et al., 2014), but not Eastern Europe, where working conditions in the nursing field are highly demanding (Hasselhorn et al., 2008; Koff, 2016; OECD/European Union, 2020). The purpose of the current research is to test the mediational effect of WFC in the job demands – job burnout link in a one-year cross-lagged design in Poland. Five different types of job demands (categorised as hindrance or challenge) were taken into account, including quantitative demands, work pace, emotional demands, cognitive demands, and demands for hiding emotions.

### Nurses in Poland

Currently, working conditions for Polish nurses are very burdensome. According to the report *Health at a Glance Europe 2020* prepared by the OECD, Poland has one of the lowest numbers of employed nurses per 1,000 inhabitants in Europe (5.1 compared to an average of 8.2 for EU countries and 17.7 for Norway, which is in the lead). The Polish Main Chamber of Nurses and Midwives (2017) forecast that by 2030 this ratio will have dropped to 3.81. The average age of Polish nurses, which is 52.2 years, is also worrying. Nearly 52% of all nurses are aged over 51 years old, while people up to 30 years old constitute only 5.5% of the workforce. Such a high average age is largely due to the emigration of younger, well-educated Polish nurses to Western European countries and little interest in studying nursing in Poland in recent years (Wyrozebska and Wyrozebski, 2014). The research shows that 53% of the surveyed nursing students declared their willingness to go abroad after completing their education, in order to work in the profession (Radosz and Paplaczyk, 2017). The motives they mention are higher earnings, better working conditions and higher prestige of the profession.

The most common system of shift work of nurses in Polish hospitals is a two-shift system including on-call time lasting 12 h, most often starting at 7.00 am or 7.00 pm (Wyrozebska and Wyrozebski, 2014). Long and irregular working hours, as well as challenging working conditions and job stress make nurses particularly prone to experiencing WFC and job burnout. A study confirms the experience of chronic work-family conflicts among 50% of nurses and episodes of conflict among another 41% (Grzywacz et al., 2006), as well as a high level of job burnout in this profession (Lloyd et al., 2002; Adriaenssens et al., 2015), also in Poland (Van der Schoot et al., 2003; Dåderman and Basinska, 2016).

## Theoretical Framework and Hypotheses

### The Effects of Challenge and Hindrance Stressors on WFC Among Nurses

Both studies on large samples of nurses (e.g., Simon et al., 2004; Leineweber et al., 2014; Unruh et al., 2016) and meta-analyses (Gonnelli and Raffagnino, 2017) confirm that prolonged high job demands lead to WFC. It is not clear, however, what types of job demands affect WFC in this professional group and how strongly (Wood and Mechaelides, 2016). A given type of job demand may or may not be aggravating and stressful, depending on whether it is perceived as challenging or hindering (Cavanaugh et al., 2000). Challenge stressors refer to those job demands that are perceived by the employee as creating opportunities for personal growth (e.g., gaining new skills, experiences, broadening horizons, reinforcing self-efficacy) and through improvement of one’s job performance give a chance for promotion or a pay rise. Hence, they can be a source of positive emotions, motivation and well-being (LePine et al., 2005; Podsakoff et al., 2007; Van den Broeck et al., 2010). In contrast, hindrance stressors concern job demands which are viewed as barriers to goal accomplishment. Handling them, at best improves the efficiency of the employee and makes them meet the imposed obligations, but is not a source of satisfaction or fulfilment *per se* (Cavanaugh et al., 2000). This typology of stressors seems to be particularly important for the nursing profession, whose scope of professional duties is very wide and contains numerous and diverse tasks (Aiken et al., 2001; Hasselhorn et al., 2008; Koff, 2016) related to quantitative demands (e.g., overtime hours, weekend work, and irregular work schedules), work pace (e.g., during medical treatment, dressings, or basic measurements), cognitive demands (e.g., acquiring new skills and qualifications, operation of medical equipment and devices, conducting education for healthy lifestyle), emotional demands (e.g., patient care, close relations with colleagues, patients and their families), demands for hiding emotions (e.g., contact with potentially infectious material, including blood, secretions, excreta, contact with chronic diseases, and death). It has been shown that the type of occupation is an important criterion for qualifying a stressor as a challenge or a hindrance. For example, nurses perceive emotional demands more as a challenge than as a hindrance. The reverse applies to quantitative demands and work pressure (Bakker and Sanz-Vergel, 2013).

Gonnelli and Raffagnino (2017) reviewed 1,180 studies on WFC, published in English and Italian in the years 2005–2017. Of these, they selected 28, which strictly concerned antecedents (and outcomes) of the conflict between work and family in groups of nurses. They found that the negative role of quantitative demands in the development of WFC is strongly supported and evident. For example, in one cross-cultural study conducted in a group of 27,603 nurses from eight countries, it was shown that quantitative demands (including intensity of work, regularity of working time, and being pressured to work overtime) are the dominating risk factors in all participating countries and they explain 13–23% of the observed variance for WFC (Simon et al., 2004). Other studies also showed that quantitative demands operationalised in different way - e.g., workload, overtime hours, weekend work and irregular work schedules – are hindering and all associated with WFC in nurses (Yildirim and Aycan, 2008; Hansen et al., 2009; Leineweber et al., 2014; Lembrechts et al., 2014; Sugawara et al., 2017).

The results of studies on the impact of emotional demands are more confused (Gonnelli and Raffagnino, 2017). In social service, there are references to two kinds of demands (Soderfeldt et al., 1996): emotionally burdensome relations with other people (e.g., in the context of long-term care, interpersonal conflicts, aggressive behaviour), and demands to observe the emotional display rules consisting in showing positive emotions and hiding the negative ones during social interactions (e.g., with a co-worker, supervisor, patient), traumatic events (e.g., emergency, patient death) and daily routines (e.g., dressings change, medical treatment). A few investigations confirm the positive relationship of WFC with emotional charge (Cortese et al., 2010), emotional dissonance (Bakker and Heuven, 2006; Ghislieri et al., 2017), aggression of patients’ experience (Peeters et al., 2004) and confrontation with death, illness, and other human suffering (Van der Heijden et al., 2008) but the correlation coefficients for emotional demands are lower than the ones for quantitative demands (Van der Heijden et al., 2008). Moreover, the results of several studies suggest that nurses are especially predisposed to perceiving emotional demands as challenge stressors (LePine et al., 2005; Bakker and Sanz-Vergel, 2013). For example, Bakker and Sanz-Vergel (2013) compared to which group of stressors (challenge or hindrance) emotional demands and work pressure are ascribed by nurses and journalists (non-social service staff). Tight deadlines are a recurring problem for journalists because newspapers and news programmes are usually distributed and broadcast daily. Hence, journalists consider time pressure as a challenge. In the case of nurses, time pressure is a hindrance because it means that there is not enough time to provide proper care to patients, which is conducive to professional fatigue and frustration. Conversely, emotional demands in nursing work (i.e., frequency of interactions with patients, and handling patient emotions and those of their family) represent “the heart of the work” and are considered a challenge (Bakker and Sanz-Vergel, 2013).

The nursing profession usually attracts people who are driven by a sense of mission and a desire to do good. McCabe et al. (2005) indicate that the most important reasons for choosing this profession are helping others, doing interesting and challenging work, and working closely with people in need. Indeed, caring for others, engaging in their problems and changing their lives can be a source of positive emotions for nurses (McQueen, 2004). Moreover, according to a qualitative survey, nurses are able to resist emotional demands and “are aware that they must actively work on their emotions” (Bolton, 2001, p. 92); therefore, we assume that emotional demands (contrary to work pace) may not necessarily be stressful, but may instead be rewarding and act as challenges for nurses. In a diary study lasting for three consecutive working weeks it was found that emotional job demands strengthened the effect of personal resources on weekly well-being, whereas work pressure undermined this effect (Bakker and Sanz-Vergel, 2013).

Cognitive demands involve confrontation with new tasks, unpredictable developments and the resolution of routine problems (Glaser et al., 2015), as well as processing multiple pieces of information at the same time, generating new ideas and making difficult decisions (Pejtersen et al., 2010). In agreement with the challenge-hindrance occupational stress model (Cavanaugh et al., 2000), they are categorised as challenge stressors. Empirical studies on cognitive demands are scarce and so far little is known about how these demands relate to the well-being of employees (Meyer and Hünefeld, 2018). To the best of our knowledge, no study has investigated the relationship between cognitive demands and WFC among nurses. Several new studies do show that cognitive demands are related positively to employee well-being – e.g., personal development (Prem et al., 2017), self-rated health and job satisfaction (Meyer and Hünefeld, 2018) – although the relation to some extent depends on the type of cognitive demand considered.

Based on the presented research reports, we expect that job demands related to hindrance (including quantitative demands and work pace) will be conducive to experiencing a conflict between work and family. In contrast, job demands related to challenge (including emotional and cognitive demands) will not lead to negative outcomes.

***H1*** *: Hindrance stressors (T1) including quantitative demands and work pace are related to high WFC (T1).*

***H2*** *: Challenge stressors (T1) including emotional demands, demands for hiding emotions and cognitive demands are related to low WFC (T1).*

### The Effect of WFC on Job Burnout

According to the JD-R model (Bakker and Demerouti, 2017), job burnout is a long-term effect of stress caused by prolonged excessive job demands and insufficient resources to deal with these job demands effectively. Job burnout consists of exhaustion and disengagement from work. Exhaustion is a response to intensive physical, affective and cognitive strain, and manifests in fatigue, weariness and a depletion of energy. Disengagement from work is expressed by distancing oneself from one’s work, and experiencing negative attitudes towards the work object, work content or one’s work in general (Demerouti et al., 2001). Maslach et al. (2001) emphasises that the risk of burnout is particularly high in the group of social service workers. Although some studies have shown that this syndrome can also affect representatives of other professions (Bakker et al., 2003), a review of studies indicates that the risk of its occurrence in employees working in direct contact with another person is particularly high (Lloyd et al., 2002).

The idea that work and family demands interfere with each other and cause strain is grounded in the Effort-Recovery model (Meijman and Mulder, 1998). Accordingly, each load involves an effort to deal with this load. The mobilisation of human strength and energy, however, is associated with high psycho-physiological costs – activation of the sympathetic nervous system, irritability, fatigue (Hockey, 1997). Therefore, the period of effort should be followed by a sufficiently long recovery. Maintaining good health and effective functioning in various spheres of life is possible when the periods of effort and recovery are balanced. Although daily work usually involves loads that are not necessarily harmful, their day after day recurrence may consequently function as a permanent source of strain. If opportunities for recovery after being exposed to a high workload are insufficient, work-related tension is transferred to non-professional fields of functioning and the employees experience conflict (Van der Heijden et al., 2008). In the long run, this conflict gradually depletes the employee’s resources required to cope with work and family demands (e.g., time, energy, mental and physical strength, abilities, equipment, social support) and job burnout occurs (Geurts and Demerouti, 2003).

The mediation effect of WFC in the job demands – burnout link was demonstrated in several studies (Geurts et al., 2003; Peeters et al., 2005), including in the nursing profession (Janssen et al., 2004; Leineweber et al., 2014; Sugawara et al., 2017). A certain limitation of these studies is their cross-sectional nature. A point is currently being raised that on the basis of a single measurement of variables at one time point, it is not possible to determine unequivocally the existence of a mediation effect (Maxwell et al., 2011). The classical method of mediation by Baron and Kenny (1986) and the Sobel (1982) test have also been long criticised, as newer and more effective methods are being used (Williams and MacKinnon, 2008; Rucker et al., 2011). We know of two cross-lagged studies on nurses confirming that the WFC mediates the negative effect of job demands on job burnout (Peeters et al., 2004) and general health (Van der Heijden et al., 2008). Both of them, however, were carried out in Western (not Eastern) European countries, where the nurses are better paid and their working conditions seem to be more comfortable (Simon et al., 2004; Koff, 2016; OECD/European Union, 2020). In addition, only one component of job burnout (exhaustion) was taken into consideration in the studies. Finally, a limited number of job demands was analysed. In the next hypothesis, we expect that:

***H3:*** *WFC (T1) mediates the effect of hindrance and challenge stressors (T1) on job burnout (T2; including exhaustion and disengagement from work).*

## Materials and Methods

### Participants

The participants (*N* = 516) were nurses employed in hospitals or clinics, in psychiatric and addiction treatment wards for children and youth (*n* = 219), as well as in social welfare homes for the chronically mentally ill, mentally disabled children, and youth (*n* = 297) in Poland. All participants were treated in accordance with the ethical guidelines of the Helsinki Declaration, and received a hard copy of the questionnaires along with a letter explaining the purpose of the study. Full confidentiality of data and anonymity were secured. Participants were asked to fill out the questionnaires and seal them in envelopes, which were subsequently collected by research assistants. Out of 750 distributed questionnaires, 591 (79%) were completed in the first step of the study (T1) and 516 (68% of the original pool) in the second stage (T2). The analysed group consisted of 431 (83.5%) women and 85 men (16.3%), between 20 and 70 years of age (*M* = 42.11, *SD* = 9.52). Work experience ranged from 1 to 45 years (*M* = 14.49, *SD* = 10.12). There were no significant differences in the distribution of *age* between the analysed occupational groups, *t*(497) = –0.48, *p* = 0.633; however, there were small but significant differences in the length of service, *t*(424.95) = –3.18, *p* = 0.002, *d* = 0.29. Nurses from social welfare homes (*M* = 13.22, *SD* = 9.46) on average had less seniority in comparison to nurses from psychiatric and addiction treatment wards of hospitals or clinics (*M* = 16.16, *SD* = 10.71).

### Measures

*Challenge and hindrance stressors* were measured with the COPSOQ II subscales (Pejtersen et al., 2010). The challenge stressors consisted of three COPSOQ II subscales measuring cognitive demands (e.g., *Does your work demand that you are good at coming up with new ideas?*), emotional demands (e.g., *Do you have to relate to other people’s personal problems as part of your work?*), and demands hiding emotions (e.g., *Are you required to treat everyone equally, even if you do not feel like it?*). Each subscale contained four items, with possible answers from 1 (Always) to 5 (Never/Hardly ever). Hindrance stressors consisted of two COPSOQ II subscales, measuring quantitative demands (four items; e.g., *How often do you not have time to complete all your work tasks?*) and work pace (three items; e.g., *Do you have to work very fast?*). Each subscale contained answers from 1 (Always or To a very large extent) to 5 (Never/Hardly ever or To a very small extent).

*Work-Family Conflict* was measured with the 5-item “work to family conflict” subscale (e.g., *The amount of time my job takes makes it difficult to fulfil family responsibilities*) developed by Netemeyer et al. (1996). It comprises answers from 1 (*I do not agree at all*) to 5 (*I fully agree*).

*Job burnout* was measured with the Oldenburg Burnout Inventory (Demerouti et al., 2001). This 16-item scale consists of two subscales for exhaustion (eight items) and disengagement from work (eight items). A 5-point response scale ranged from 1 (I completely disagree) to 5 (I completely agree).

### Analytical Procedures

Prior to the verification of the hypotheses, descriptive statistics were calculated and a correlation analysis was carried out. In order to determine the factor accuracy and to estimate the parameters of fit, a confirmatory factor analysis (CFA, first and second order) of the tools used in the structure proposed by its authors was also carried out. The structure of the tools was confirmed as judge by the models’ fit indices (Supplementary Table 1A). By good model fit we understood the values between 1.00 and 3.00 for CMIN (χ^2^/*df*), values above 0.95 for CFI, values below 0.06–0.08 for RMSEA, values for SMRM less than 0.06–0.08, and PClose above 0.05 (Hu and Bentler, 1999). As an acceptable fit we assumed CMIN values between 3 and 5, RMSEA below 0.08–0.10, PClose between 0.01 and 0.05, for SMRM it was a range of 0.08 and 0.10, and for CFI values between 0.90 and 0.95 (Sharma, 1996; Kline, 2005; Byrne, 2010). We did not expect, however, the non-significant coefficient for χ^2^ test which indicates a good fit of the data to the model but in general is very difficult to achieve especially for the larger sample sizes, as in here.

For the main part of the analysis, structural equation modelling (SEM) was applied. Challenge stressors and hindrance stressors (T1), work-family conflict (T1) and job burnout (related to exhaustion and disengagement from work; T2) were introduced into the model. The following were tested: (1) the direct effects of challenge stressors (**CS**) and hindrance stressors (**HS**) on job burnout (**JB**); (2) the indirect effects of work-family conflict (**WFC**) on CS/HS – JB links.

## Results

### Preliminary Analysis Including Descriptive Statistics

We began the analytical work with calculations of descriptive statistics for the main study constructs (Table 1) and also for the main study constructs and sociodemographics (Supplementary Table 2A). Then, we continued with SEM using SPSS ver. 26 and Amos ver. 26. Prior to conducting any analysis, all data was prechecked in order to detect potential violations of necessary preconditions (e.g., normality of distributions, linearity, but also lack of outlying cases, analysis of patterns of missing data including their randomness) to run particular statistical tests. We did not detect any significant departures. According to the results of basic correlational analysis, our main study constructs were significantly intercorrelated, mostly in postulated directions (Table 1), thus setting up a good baseline for more advanced hypotheses testing.

**TABLE 1:** Pearson’s r correlational coefficients for main study constructs, *N* = 516.

	(1)	(2)	(3)	(4)	(5)	(6)	(7)	(8)
1. Quantitative demands	−							
2. Work pace	0.37***	−						
3. Cognitive demands	0.19***	0.55***	−					
4. Emotional demands	0.25***	0.46***	0.70***	−				
5. Demands for hiding emotions	0.26***	0.38***	0.31***	0.32***	−			
6. Work - family conflict	0.49***	0.38***	0.28***	0.34***	0.27***	−		
7. Exhaustion (time 2)	0.23***	0.16***	0.05	0.12**	0.17***	0.19***	−	
8. Disengagement (time 2)	0.19***	0.13**	0.02	0.03	0.12**	0.19***	0.66***	–

* p < 0.05, ** p < 0.01, ***p < 0.001.

### Structural Equation Modelling

**FIGURE 1:**
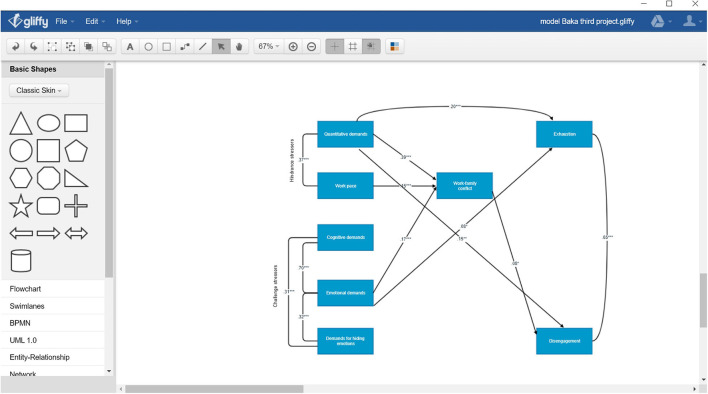
Hypothesised model for the link between various job demands and job burnout in two forms including additional mediational effects. Non-significant paths are not included. Standardised coefficients are presented. Some coefficients of covariance were omitted for clarity. The magnitude of intercorrelations between constructs could be found in Table 1.

We started the main analysis with the first model (M1) including mediational effects as postulated in hypotheses H1–H3. M1 was a model with zero degrees of freedom so we could not judge its overall fit. We were able, however, to test the significance of particular paths and mediational effects. Upon inspecting the regression paths, we trimmed M1 in a step-by-step procedure by removing non-significant paths. The final Model (M2) is presented in Figure 1. M2’s quality of fit was judged based on before mentioned criteria. M2 had an excellent fit according to the values of the indices: χ^2^(10) = 16.08, *p* = 0.098, χ^2^/df = 1.61, RMSEA = 0.03 [0.00, 0.06], PClose = 0.780, CFI = 0.99, TLI = 0.99, SMRM = 0.03. All regression paths were significant (at least at *p* < 0.05). None of the correlations within particular constructs were exceeding a value of *r* = 0.85, which would suggest potential discriminative issues (Kline, 2005). Also, no re-specifications based on error covariances were added since M2 had already an excellent fit. The first set of hypotheses (H1 and H2), which was tested by examining regression paths was partly confirmed. As seen in Figure 1, quantitative demands (β = 0.39, *B* = 0.58, *p* < 0.001) and work pace (β = 0.15, *B* = 0.26, *p* < 0.001), which together constitute HS, were related to higher WFC. The results for H1 allied with the outcomes of correlational analysis (Table 1). CS, however, was only partly related to WFC (H2). The paths from demands for hiding emotions and cognitive demands were excluded from the final M2 (as M2 is based on M1), since they were not significant predictors of WFC (*B* = 0.08, *p* = 0.106 and *B* = 0.02; *p* = 0.814, respectively; based on M1). Emotional demands were significantly related to WFC, but the direction of the relation was positive in M2 (β = 0.17, *B* = 0.23, *p* < 0.001), and therefore opposite to that predicted in H2. Both the results of the SEM and correlational analysis do not support H2.

The mediational hypothesis was tested in the next step using just identified model (M1). The results confirmed that WFC plays the role of mediator in job demands – job burnout link, depending on the type of job demands, as well as components of job burnout. In the case of HS, WFC mediated the effect of quantitative demands [*ab* = 0.001, BCa 95% CI (0.000, 0.002), *p* = 0.023]^1^ and work pace [*ab* = 0.001, BCa 95% CI (0.000, 0.001), *p* = 0.019] on disengagement from work but not on exhaustion [*ab* = 0.001, BCa 95% CI (0.000, 0.002), *p* = 0.176 and *ab* = 0.000, BCa 95% CI (0.000, 0.001), *p* = 0.112, respectively]. Thus, for hindrance stressors, H3 was partly confirmed. In the case of CS, the role of WFC as mediator was confirmed only for the relationship between emotional demands and disengagement from work [*ab* = 0.000, BCa 95% CI (0.000, 0.001), *p* = 0.017], but not for the exhaustion [*ab* = 0.000, BCa 95% CI (0.001, 0.001), *p* = 0.146]. There were no significant mediational effects for the link between cognitive demands, demands for hiding emotions and two components of job burnout (as analysed in M1, which included all possible paths). Thus, overall H3 was only partly confirmed (only for mediational effects related to disengagement from work).

## Discussion

The article discusses the longitudinal relationship between various types of job demands occurring in nursing work environments and two components of job burnout (i.e., exhaustion and disengagement from work), with the mediating effect of WFC. Five different job-related risks factors (i.e., quantitative demands, work pace, emotional demands, cognitive demands, and demands for hiding emotions) were taken into account and classified into two groups of stressors: challenge and hindrance. Based on theoretical premises (McQueen, 2004; McCabe et al., 2005) and research results (LePine et al., 2005; Bakker and Sanz-Vergel, 2013; Gonnelli and Raffagnino, 2017), it was assumed that emotional demands, cognitive demands and demands for hiding emotions can be considered as challenges in the nursing profession and, therefore, should not lead to negative outcomes. Contrary to them, quantitative demands and work pace usually mean that nurses do not have time to provide patients with the appropriate care, lead to role conflicts, and are therefore perceived by nurses as hindering.

The hypotheses regarding the different impact of challenge and hindrance stressors on WFC (H1, H2) were only partially confirmed. As predicted (H1), hindrance stressors (T1, especially quantitative demands) were related to high work-family conflict (T1) and job burnout (T2). It turned out, however, that challenge stressor related to emotional demands also lead to negative outcomes (higher WFC and job burnout). This result (inconsistent with H2) shows that the provision of long-term care for other people can be perceived as burdensome and limiting, even among medical staff, where help is included in the “nature” of the profession. The remaining challenge stressors related to cognitive demands and demands for hiding emotions were not associated with negative outcomes. Unexpected relationship of emotional demand with WFC and job burnout can be explained in several ways. First, although the authors emphasise that challenge stressors can be a source of positive outcomes (e.g., LePine et al., 2005; Podsakoff et al., 2007), it does not mean that they are not cognitively and emotionally aggravating. In a more recent meta-analysis of 31 studies, Mazzola and Diheltorst (2019) showed that both hindrance and challenge stressors are positively correlated with psychological strain (the mean correlations were ρ = *0.29* for CS and ρ = *0.36* for HS) and physical strain (ρ = 0.24 for CS and ρ = 0.38 for HS). This strain can be the result of experiencing a role conflict. Some previous studies have shown that emotional demands in groups of nurses, measured by means of various indicators including emotional charge (Cortese et al., 2010), emotional dissonance (Bakker and Heuven, 2006), and confrontation with traumatic events (Van der Heijden et al., 2008), were associated with high work-family conflict. It may be that individual differences (Wilson, 2012), appraisals (Li et al., 2020), access to high job resources (Dawson et al., 2016), and the level of professional competences (Horan et al., 2020), play a greater role in classifying a given stressor to the challenge or hindrance group, than occupation-related specificity. Perhaps not without significance is also the fact that the studied sample was dominated by women who, according to the results of the research, experienced lower levels of challenge stressors (Mazzola and Diheltorst, 2019).

Thus, individuals can appraise stressors differently, based on any number of internal and external variables. Moreover, perceptions of stressors tend to have considerable within-person variability. This means that employees can be very positive about their work one day and much less positive the next day. Thus, it is entirely possible that an employee may perceive certain tasks in their job as a challenge on one day and feel that the same tasks are hindering on another day (Horan et al., 2020). Some researchers emphasise that stability during challenge and hindrance stressor appraisals is also important (Rosen et al., 2020). For example, when challenge stressors were stable week-to-week, individuals were better able to anticipate them, relative to when they fluctuated. As a result of anticipating stressors, individuals appraised them as challenging and ultimately experienced less overall stress. Individuals who experienced more fluctuations in challenge stressors, however, assessed them as more hindering, exhibited worse task performance and reported greater subjective stress due to lower stressor anticipation (Rosen et al., 2020).

Regarding the mediational effect, the results confirmed that WFC mediates the harmful impact of job-related risk factors on job burnout, but mainly in relation to hindrance stressors (H3) and one of job burnout’ component. HS (included quantitative workload and work pace, T1) led to high disengagement from work (T2) via increased work-family conflict (T1). In the case of challenge stressors (T1), WFC (T1) mediated only the effect of emotional demands (not cognitive demands and demands for hiding emotions) on disengagement from work (T2). WFC did not related to exhaustion (T2). In general, the results are consistent with the JD-R model, but only partially. According to the JD-R model, different kinds of job demands result in high job burnout, however, their negative effect should be stronger for exhaustion than for disengagement from work. Based on the results obtained in initial study on the JD-R model, Demerouti et al. (2001) postulated even, that job demands are related to exhaustion component of job burnout, whereas low job resources are related to disengagement from work. The findings of our study indicate, however, that after including WFC as a mediator in the model, job demands affect disengagement from work, not exhaustion. Such distancing from work may be a way of coping with highly burdensome demands (e.g., entering into close and emotionally exhausting relationships with patients) and chronic work-family interference. In other words, by avoiding work commitments and emotional withdrawal, overworked nurses can save stress-depleted resources and preserve "good" health (Hobfoll, 2011).

It is worth mentioning a few contributions of our study. First of them, it found that not all types of job demands have the same negative consequences for nurses over a one-year time frame. While the detrimental function of job-related factors classified as hindrance was confirmed, it turned out that the impact of challenge stressors is more complex. Although high emotional demands resulted in an impairment of nurses mental health after one year, cognitive demands and demands for hiding emotions are not associated with negative outcomes. It is possible that these types of demands are less burdensome for nurses, or they may have other beneficial effects that neutralise the harmful ones. The next contribution refers to the confirmation of the mediational effect of WFC in one-year cross-lagged study. This effect was mainly related to hindrance stressors and was observed only in the case of disengagement from work. And last but not least, our study supported validity of the Job Demands-Resources and the Effort-Recovery models also in Eastern Europe.

### Limitations and Future Research Direction

This research is not without limitations. Although the cross-lagged study with a year interval design can clarify the direction between job demands, job resources and mental health, it is not justified to draw valid causal inferences (Taris and Kompier, 2003). Conducting a quasi-experimental study that manipulates job demands and job resources, however, would be difficult to perform and would raise serious ethical issues. Another limitation is the number of measurements. In this two-wave study, hindrance/challenge stressors and WFC were checked at the same point of measurement. It would be optimal to conduct a three-wave study, with a separate measurement for the mediator and dependent variable (Hakanen et al., 2008). When considering generalisability, it should be noted that the results of this study were obtained from a sample of nurses. The observed regularities relate to this profession only and should not be generalised to other occupations and market sectors. A final issue is the gender disproportion in the research sample. Women were overrepresented, because the number of women in the nursing field is significantly greater. For the male population, in traditionally typical male occupations, the results would be perhaps different.

Another issue is that the research was conducted during the COVID-19 pandemic; hence, some responses (e.g., related to workload and exhaustion) may be biassed by the specificity of the current situation. During a pandemic, the organisation of work and the level of job demands are different from traditional ones. For example, nurses face a great amount of unusual job-related stressors, including staff shortages, insufficient equipment, inadequate protection from contamination, risk of infection, work overload, social stigmatisation, isolation, lack of contact with their families, as well as lack of consistent information about the spread of the virus, its contagiousness and the effectiveness of ways of prevention (Fiorillo and Gorwood, 2020). These unusual job conditions may have a significant impact on the results obtained. Additionally, no moderation effects were controlled in the study, both with respect to job characteristics and to personality variables. It seems that rich job resources (such as leadership, social support, or job control), as well as personality traits (such as hardiness or self-efficacy) may buffer the detrimental effects of job stressor and WFC on health of nurses.

Apart from some limitations, the study adds to knowledge by the detailed investigation of the links between various types of stressors and two forms of job burnout throughout work-family interference with focus on potentially moderating effects of personal and leadership resources in an important context of nursing profession in Eastern Europe. In future studies, it would be worth investigating the role of other types of job and personal resources in coping with strain, e.g., meaning of work (Jourdain and Chênevert, 2010) or optimism (Garrosa et al., 2011). Those that relate to specific personal resources in a professional context would be particularly useful (e.g., occupational hardiness, occupational resilience). As suggested by some authors, this kinds of specific resources are particularly effective in reducing stress (de Jonge et al., 2000).

## Data Availability Statement

The raw data supporting the conclusions of this article will be made available by the authors, without undue reservation.

## Ethics Statement

The studies involving human participants were reviewed and approved by research committee at the Cardinal Stefan Wyszyński University in Warsaw on June 2020 (number: KEiB 31/2020). The patients/participants provided their written informed consent to participate in this study.

## Author Contributions

ŁB: conception and design of the study and organisation of the database. MP: statistical analysis. ŁB and MP: first draft of the manuscript and preparation of the manuscript. Both authors contributed to the article and approved the submitted version.

## Conflict of Interest

The authors declare that the research was conducted in the absence of any commercial or financial relationships that could be construed as a potential conflict of interest.

## Publisher’s Note

All claims expressed in this article are solely those of the authors and do not necessarily represent those of their affiliated organizations, or those of the publisher, the editors and the reviewers. Any product that may be evaluated in this article, or claim that may be made by its manufacturer, is not guaranteed or endorsed by the publisher.

## Footnotes

1 ab – the coefficient of indirect effect; BCa 95% CI - bias corrected and accelerated bootstrapp confidence intervals of 95 percent.
