# Supplementary material for: Towards Better Understanding of the Harmful Impact of Hindrance and Challenge Stressors on Job Burnout of Nurses. A One-Year Cross-Lagged Study on Mediation Role of Work-Family Conflict
Source: Front Psychol. 2021 Sep 17;12:696891. doi: 10.3389/fpsyg.2021.696891 (PMC8484705; doi:10.3389/fpsyg.2021.696891)
Supplement: Supplementary file 1 [file Data_Sheet_1.DOCX]

**Appendix**

Table 1A

*Model Adequacy and Goodness of Fit Indices of the Models Tested Using First and Second Order Confirmatory Factor Analysis*

| Models | Χ^2^ | *df* | *p* | Χ^2^*/df* | RMSEA | PClose | SRMR | CFI | AIC | β  range  absolute  value |
| --- | --- | --- | --- | --- | --- | --- | --- | --- | --- | --- |
|  |  |  |  |  |  |  |  |  |  |  |
| **Model – “Hindrance stressors”**  **– First order** | 94.39 | 41 | < .001 | 2.30 | .05 | 465 | .04 | .97 | 166.39 | [.52, .81] |
| **Model – “Hindrance stressors”**  **- Second order** | 77.64 | 41 | < .001 | 1.89 | .06 | .312 | .05 | .97 | 149.64 | [.41, .86] |
| **Model - ”Challenge stressors”**  **- First order** | 320.04 | 51 | < .001 | 6.63 | .10 | .000 | .07 | .85 | 398.04 | [.37, .80] |
| **Model - ”Challenge stressors”**  **- Second order** | 320.04 | 51 | < .001 | 6.28 | .10 | .000 | .07 | .95 | 398.04 | [.37, 1.00] |
| **Model - ”Challenge stressors”**  **- Second order with modifications** | 164.22 | 46 | < .001 | 3.57 | .07 | .002 | .05 | .93 | 252.22 | [.37, 1.00] |
|  |  |  |  |  |  |  |  |  |  |  |

*Note.* CFA = RMSEA = root mean square error of approximation; PClose = *p* of Close Fit; SRMR = standardized root mean square residual; CFI = comparative fit index; AIC = Akaike information criterion. The respecifications of models were achieved based on error covariance modification indices.

Table 2A

*Pearson’s r Correlational Coefficients for Main Study Constructs and Basic Sociodemographics,* N *= 516*

|  | Gender  1-F | Age | Education | Seniority at work | Seniority at work  sqrt^a^ |
| --- | --- | --- | --- | --- | --- |
|  |  |  |  |  |  |
| 1. Quantitative demands | .03 | -.05 | **.13**** | .01 | .04 |
| 2. Work pace | .08 | 0 | .05 | .02 | -.01 |
| 3. Cognitive demands | **.11*** | **-.11*** | **.21***** | -.05 | -.05 |
| 4. Emotional demands | **.20***** | .00 | **.17***** | .03 | .00 |
| 5. Demands for hiding emotions | .06 | -.04 | .01 | .01 | **.09*** |
| 6. Work – family conflict | .04 | -.02 | .03 | .07 | -.01 |
| 7. Exhaustion (time 2) | .00 | -.01 | **-.09*** | .00 | -.06 |
| 8. Disengagement (time 2) | -.06 | -.01 | -.08 | .03 | .00 |
|  |  |  |  |  |  |

*Note.* * *p* < .05, ** *p* < .01, ****p* < .001.
